# From structure to sequence: A multinomial processing tree model of syntactic encoding

**DOI:** 10.3758/s13423-026-02958-9

**Published:** 2026-07-29

**Authors:** Jeremy D. Yeaton, Grant M. Walker, Danielle Fahey, William Matchin, Julius Fridriksson, Gregory Hickok

**Affiliations:** 1 Department of Language Science, University of California, Irvine, Irvine, CA 92697 USA; 2 Department of Neurological Surgery, University of California, Davis, Davis, CA 95616 USA; 3 Department of Cognitive Science, University of California, Irvine, Irvine, CA 92697 USA; 4 Department of Communicative Disorders, University of Alabama, Tuscaloosa, AL 35487 USA; 5 Department of Communication Sciences and Disorders, University of South Carolina, Columbia, SC 29208 USA

**Keywords:** Computational modeling, Language production, Neuroimaging, Syntax, Psycholinguistics

## Abstract

Two-stage models of grammatical encoding posit that sentence production unfolds in two sequential steps: the construction of a hierarchical syntactic structure, followed by its linearization into a sequence suitable for articulation. While widely accepted in psycho- and neurolinguistics, such models lack formal computational implementations. Here, we introduce a novel multinomial processing tree (MPT) model that operationalizes this two-stage framework to explain syntactic error patterns in individuals with aphasia. Drawing on discourse samples annotated for distinct error types, we fit an MPT model to estimate individual abilities at each processing stage. These ability estimates correlated with observed error rates and localized to distinct neural substrates: hierarchical encoding ability was linked to the posterior superior temporal sulcus and parietal cortex, linearization to posterior inferior frontal regions, and omission-related processes to more dorsal frontal regions and the underlying white matter. Our findings support a neurocomputational dissociation between hierarchical and linear stages of grammatical encoding, aligning with prior lesion-symptom mapping and theoretical accounts of expressive agrammatism and paragrammatism. This work represents the first computational instantiation of a two-stage model of syntactic production, bridging formal modeling and lesion analysis to advance our understanding of the architecture and breakdown of grammatical encoding.

## Introduction

Two-stage models of grammatical encoding are among the most widely accepted frameworks in psycho- and neurolinguistics (Bock & Levelt, 1994; Ferreira et al., 2018; Matchin & Hickok, 2020; Yeaton, 2025), yet they have never been formally implemented as computational models of sentence production. Without a computational instantiation, it is impossible to derive quantitative, individual-level estimates of processing ability at each stage, or to make precise quantitative predictions about the neural substrates of each stage. Here we address this gap by introducing a multinomial processing tree (MPT) model that operationalizes the two-stage framework as a generative process and fits it to behavioral data from individuals with aphasia.

Under a two-stage model of grammatical encoding, sentences to be expressed begin as some unstructured semantic or message-level information (Krauska & Lau, 2023; Matchin & Hickok, 2020). Based on this message, the first stage selects appropriate lexical information and constructs an unordered *hierarchical* tree containing the relevant syntactic and relational information. The second stage of grammatical encoding interprets this abstract hierarchy and converts it into a *linear* sequence to be deployed by the speech-motor system. Different iterations of the two-stage model use different names for the two stages: Bock and Levelt (1994) dub them functional & positional; Krauska and Lau (2023) call them representation & linearization. In this work we will use the terms hierarchical and linear, following Matchin and Hickok (2020), referring to this iteration of the two-stage model as HiLine (Hickok, 2025).

### Expressive syntactic deficits in aphasia

Two major expressive syntactic syndromes are attested in post-stroke aphasia: agrammatism & paragrammatism (Goodglass, 1968; Jakobson, 1956; Kean, 1977; Kleist, 1914; Matchin et al., 2020). Agrammatism has been studied extensively since at least the late 19th century (e.g., Kussmaul, 1877; Pick, 1913), and paragrammatism was first systematically described by Kleist (1914). Agrammatism is typified by the simplification or reduction of syntactic structure by the omission of functional morphology or semantically light lexical items, leading to its characterization as “telegraphic” (Goodglass, 1968; Kean, 1977). Paragrammatism, on the other hand, is characterized by insertions and substitutions of grammatical content, resulting in “sentence monsters” (Kleist, 1914).

Although agrammatism has traditionally been associated with nonfluent aphasic syndromes (e.g., Broca’s aphasia) and paragrammatism with fluent syndromes (e.g., Wernicke’s, conduction aphasia) (Butterworth & Howard, 1987; Goodglass, 1968), the present work focuses on the syntactic error patterns themselves, independent of clinical syndrome labels. Critically, the distinction between these two error types maps naturally onto the two stages of the HiLine model: agrammatic errors, characterized by the omission of functional morphology and telegraphic output, are consistent with a breakdown at the linearization stage, where functional morphemes are implemented; paragrammatic errors, characterized by substitutions, insertions, and structural anomalies, are consistent with a breakdown at the hierarchical stage, where relational structure is built (Krauska & Lau, 2023; Matchin & Hickok, 2020). This mapping provides both a theoretical motivation for the error taxonomy used here and a basis for predicting distinct neural correlates of the two error types.

Recent work in aphasiology has proposed that breakdowns can occur independently at either of the two stages of grammatical encoding (Krauska & Lau, 2023; Yeaton et al., 2026), paving the way for investigations into a dissociation at both the computational and neural levels. Lesion-deficit correlation studies have found that agrammatism is associated with damage to the frontal cortex, including Broca’s area (den Ouden et al., 2019; Wilson et al., 2010), while paragrammatism is associated with posterior temporal-parietal lesions (Casilio et al., 2024; Gleichgerrcht et al., 2021; Matchin et al., 2020). These neural dissociations provide converging evidence that the two error types reflect distinct underlying computational mechanisms, and motivate the use of a model-based approach to quantify each individual’s deficit at each stage of processing. It has further been proposed that syndromes affecting expressive syntax are attributable to breakdowns at these two stages: agrammatism is due to a breakdown in the linearization stage, and paragrammatism is due to a breakdown in the hierarchical stage (Yeaton et al., 2025).

### Multinomial processing tree models

Multinomial processing tree (MPT) models describe the processing steps leading to some observed categorical behavior (Batchelder, 1998; Batchelder & Riefer, 1999). The steps can be formalized as a binary branching tree, where each bifurcation is associated with a parameter representing the probability of successful processing at that step, and each leaf node is associated with a response type. The probability of each response type is easily calculated as a product of the branches leading from the root node to the leaf node(s) of interest, and summing these products if there are multiple leaf nodes of the same type. MPT models have been constructed to explain performance on a wide variety of psychological tests (see Erdfelder et al., 2009, for a review). MPT models can then be fit to behavioral data to produce ability estimates for the different stages of the generative process. Of most relevance are recent MPT approaches to model picture naming error commission in both post-stroke aphasia (Walker et al., 2018) and primary progressive aphasia (Petroi et al., 2021). The ability estimates from these MPT models of naming behavior have been shown to reliably predict performance on other linguistic assessments as well as produce meaningful lesion correlates (Walker, 2021).

### Kinds of syntactic errors

A novel approach for characterizing syntactic output in aphasia was recently developed by Yeaton et al. (2026). Based on their interpretation of the HiLine model, they created a coding scheme to be used to characterize utterances produced in discourse according to the types of errors they contain. The mapping of error types to processing stages follows directly from what is known about what each stage computes. Because the hierarchical stage is responsible for building relational structure – encoding argument relations, selecting appropriate syntactic frames, and joining lexical items into a hierarchically organized representation – breakdowns at this stage are expected to produce errors such as subcategorization violations, exchange errors, fusion errors, and insertion of lexical items into structurally anomalous positions (Bock & Levelt, 1994; Matchin & Hickok, 2020). By contrast, because functional morphology (e.g., inflectional affixes, determiners, auxiliaries) is implemented during the linearization stage when the abstract hierarchy is mapped onto a sequential form, omission of multiple functional morphemes and telegraphic output are expected to reflect linearization-stage breakdown (Bock & Levelt, 1994; Ferreira et al., 2018). Very briefly, utterances with multiple omissions of functional morphology are characterized as linearization errors, while ungrammatical utterances due to insertions, substitutions, or transpositions are characterized as hierarchical errors. Grammatically well-formed utterances are marked as grammatical. Transcripts of people with aphasia (PWA) retelling the story of Cinderella (MacWhinney et al., 2011) were annotated according to this coding scheme.

The categorical data produced by this coding scheme are an ideal candidate for a syntactic extension of MPT modeling of language errors: observed syntactic errors are attributable to breakdowns at the respective stages of the computational process. Utterances can be classified according to the types of errors they contain, which results in the necessary leaf nodes for the MPT model. Importantly, this approach allows us to move beyond holistic clinical impressions of a patient’s speech and instead derive quantitative, stage-specific ability estimates for each individual, which can then be used to examine the neural bases of each processing stage through lesion mapping.

In this work, we have developed a cognitive psychometric MPT model which generates ability estimates for the two stages of the HiLine on the basis of observed distributions of syntactic error types. We find that the ability estimates generated by this model produce meaningful lesion correlates, and we discuss the relevance of MPT modeling to advancing our understanding of both healthy and disordered language, and how this work might be extended to provide a more fine-grained picture of the syntactic computation system.

## Methods

### Input data

As input data, we used the annotated discourse data from Yeaton et al. (2026). We thus have error-rate information for the 79 people with aphasia who participated in that study. Each participant produced a variable number of sentences, and each sentence was scored as either correct or one of four possible error types (i.e., hierarchical, linear, mixed, omission), yielding five categories of frequency counts per participant.

- Grammatical: Utterances that are well-formed sentences or phrases in standard American English
- Hierarchical processing error: Utterances containing multiple mismatching morphemes, insertion of lexical items into incorrect syntactic nodes (subcategorization violation), incorrect argument order (but no missing elements), omission of branching structures or lexical items, omission of (lexical) verb or verb phrase (but not copula). These features are diagnostic of hierarchical-stage breakdown because the hierarchical stage is responsible for constructing the relational structure that governs argument selection, subcategorization, and the hierarchical organization of phrases (Bock & Levelt, 1994; Matchin & Hickok, 2020); errors at this stage therefore manifest as structural anomalies rather than missing morphological material
- Linearization processing error: Utterances missing multiple functional morphemes. Functional morphology (e.g., inflectional affixes, auxiliaries, determiners) is implemented during the linearization stage when the abstract hierarchy is converted to a sequential form (Bock & Levelt, 1994); accordingly, the omission of multiple such elements is taken as evidence of a breakdown specifically in linearization rather than in hierarchical structure-building
- Mixed hierarchical & linear errors (HL): Utterances that contain multiple missing morphemes as well as any of the markers of hierarchical processing errors above
- Omission: Ungrammatical utterance where H/L is indeterminate due to single omission of a functional morpheme

Single omission errors are included as a separate category due to the difficulty of determining whether they arise from a breakdown in hierarchical or linearization processing. For example, in an utterance like “*two boy walk*”, where the plural*-s* is absent from the noun, the missing morpheme could be a linearization error resulting in the omission, or due to a failure to establish the proper dependency relations during the hierarchical phase. Additional detail and validation of this coding scheme are presented in Yeaton et al. (2026).

**Fig. 1:**
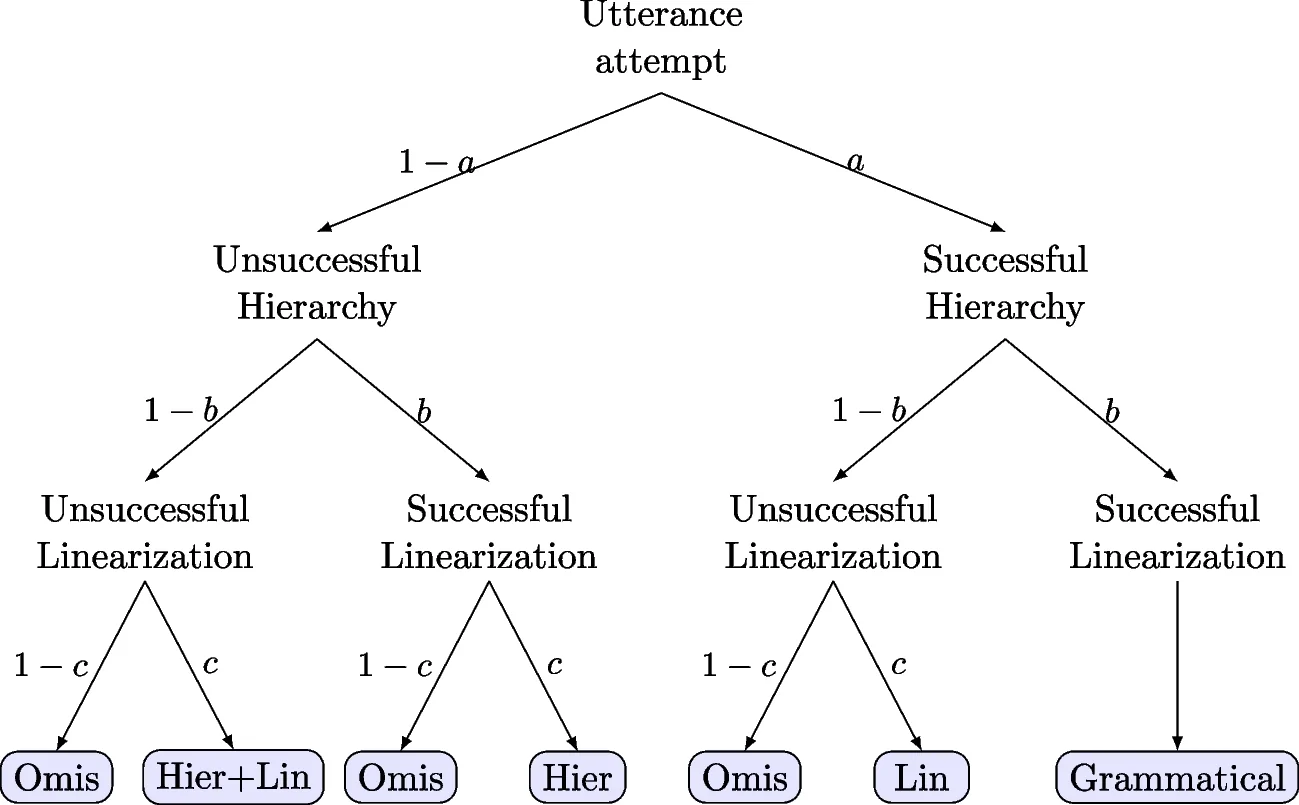
Multinomial processing tree model. *a* represents the ability estimate for producing well-formed hierarchical structure, *b* represents the ability estimate for correctly linearizing a hierarchical structure, and *c* represents the probability that some error at either the hierarchical or linear level will present as an omission

### Participants

The original discourse data were collected at the University of South Carolina. Participants were drawn from a database of individuals with chronic, post-stroke aphasia who have completed testing for various studies conducted at the University of South Carolina and the Medical University of South Carolina over the last 15 years. All were native speakers of English, had suffered an ischemic stroke to the left hemisphere at least six months prior to the study, and presented with language difficulties (all participants had presented with aphasia in the acute phase following their stroke, which formed the basis of their enrollment in the study, and most participants were classified as aphasic according to the WAB-R [Kertesz, 2007], though some scored outside of the aphasic range by the time of examination). From this database, 79 participants were selected (mean age = 59.7 years old (range 29–80 years old), 31 women). Participants were diagnosed with the following aphasia types: 21 with anomia, 25 with Broca’s aphasia, 18 with conduction aphasia, two with global aphasia, one with transcortical motor aphasia, one with transcortical sensory aphasia, and five with Wernicke’s aphasia. Six participants were classified as not aphasic according to the WAB-R. Fifty-one of the included participants were selected because of their inclusion in Matchin et al. (2020). Selection for that study was based on the amount of intelligible speech output recorded during their retelling of the story of Cinderella; see below). The remaining 28 participants were selected from the database based on the availability of imaging data, and a transcribed Cinderella discourse sample with at least ten intelligible, non-filler utterances (Yeaton et al., 2026). All procedures were approved by the internal review board at the University of South Carolina (Pro00053559), informed consent was obtained, and data were de-identified.

### Model

We constructed a multinomial processing tree (MPT) model based on the five categories of response types (Fig. 1). The model included three latent parameters: (a) the probability of constructing the correct hierarchical representation of a sentence, (b) the probability of constructing the correct linearized form of a hierarchical representation, and (c) the probability of anticipating and suppressing an omission error. The probability of observing each response type was derived from the MPT model equations (Eq. 1). We used a probit model for each latent parameter, sampling from a prior standard normal distribution (0, 1), and applying the normal cumulative distribution function to convert samples to the probability scale, [0, 1].

1 $$\begin{aligned} \begin{aligned} p(\textsc {Grammatical})&= a \cdot b \cdot c\\ p(\textsc {Hierarchical error})&= (1-a) \cdot b \cdot c\\ p(\textsc {Linearization error})&= a \cdot (1-b) \cdot c\\ p(\textsc {Mixed error})&= (1-a) \cdot (1-b) \cdot c)\\ p(\textsc {Omission error})&= (1-a) \cdot b \cdot (1-c) + a \cdot (1-b)\\&\quad \cdot (1-c) + (1-a) \cdot (1-b) \cdot (1-c) \end{aligned} \end{aligned}$$

#### Justification of prior distributions

The standard normal prior distribution in a probit model corresponds to a uniform prior distribution over the [0, 1] interval. That is, before observing data, all probability values are assumed to be equally likely.

#### Model fitting

To estimate the latent parameters *a*, *b*, and *c* for each participant, we fit the MPT model to each participant’s observed distribution of response types. Intuitively, the fitting procedure works by searching for the values of *a*, *b*, and *c* that make the probabilities predicted by Eq. 1 best match that participant’s actual proportions of grammatical responses, hierarchical errors, linearization errors, mixed errors, and omissions. This search is carried out using a Bayesian sampling approach: rather than settling on a single best-fitting value for each parameter, the procedure generates a large collection of plausible parameter values (a posterior distribution) that are consistent with the observed data. Parameters that produce predictions closer to the observed data are sampled more often, while implausible values are sampled rarely or not at all. The result for each participant is a distribution of credible *a*, *b*, and *c* estimates rather than a single point estimate, which allows uncertainty in the parameter values to be propagated through subsequent analyses.

We used JAGS software (Plummer et al., 2003) to implement Gibbs sampling of latent variables. We initiated four chains with random draws from a standard normal distribution, each beginning with 100 burn-in samples followed by 1000 samples of the posterior distribution, for a total of 4000 posterior samples per latent probability.

#### Convergence

The maximum R.hat statistic for any estimated latent probability was 1.0036, well below the 1.01 threshold for convergence.

#### Posterior predictive checks

The minimum posterior predictive density across all participants and response types was 5.65e$^{-7}$, and the average posterior predictive density for all participants and response types was .0202. All observations fell within the predicted range of values based on the fitted model.

#### Model comparison

We compared the MPT model against a simple multinomial model in which each response type rate is assigned its own latent variable, yielding four free parameters and a fifth that must sum to one. Whereas in the MPT model, the parameter estimates represent latent abilities, in the simple multinomial model, the parameters simply represent the probabilities of committing each of the error types, without considering that there may be some underlying relationship between the types of errors committed. The multinomial model parameters were estimated in the same way as the MPT model, using a probit model for latent probabilities and Gibbs sampling with four chains of 1000 posterior samples with 100 burn-in samples. We compared the deviance information criterion (DIC) between the models; a lower DIC indicates the preferred model.

Although we anticipated that the MPT model might not outperform the multinomial model on raw fit – the multinomial model has more free parameters and places no theoretical constraints on the relationships between error types – the value of the MPT approach lies not in fit alone but in the interpretability of its parameters. The latent abilities *a*, *b*, and *c* have explicit theoretical meanings grounded in the two-stage architecture, and their validity can be assessed independently through their lesion correlates. We therefore proceed to lesion mapping based on the MPT parameters rather than the multinomial error rate estimates.

We further carried out two-sided Pearson correlation tests between MPT ability estimates and observed error rates. *p*-values were Bonferroni-corrected for multiple comparisons.

### Lesion–parameter mapping

High-resolution magnetic resonance imaging (MRI) data (T1- and T2-weighted images) were collected at the University of South Carolina and the Medical University of South Carolina on a 3T Siemens Trio scanner with a 12-element head coil. T1-weighted MRI images were collected using an MP-RAGE sequence, voxel dimensions 1 mm$^3$, 256 x 256 matrix, 9$^\circ$ flip angle, TR 2250 ms, either 160 slices with inversion time of 900 ms and echo time of 4.52 ms, or 192 slices with inversion time of 925 ms and TE of 4.15 ms with parallel imaging (GRAPPA = 2, 80 reference lines). T2-weighted MRI images were collected using a sampling perfection with application optimized contrasts with a different flip angle evolution sequence (3D-SPACE). This 3D turbo spin echo scan has 192 slices 1 mm thick, TR of 2800 ms, TE of 402 ms, variable flip angle, 256 x 256 matrix, with parallel imaging (GRAPPA = 2, 120 reference lines). Lesions were demarcated onto each participant’s T2 image by an expert neurologist or an expert cognitive scientist, each blind to the behavioral data. Lesion maps were then aligned to the high-resolution T1 image. Lesions were replaced with the corresponding brain structure from the intact hemisphere, and this image as well as the lesion map in participant space were subsequently warped to Montreal Neurological Institute (MNI) space (Nachev et al., 2008) using SPM12 (Ashburner & Friston, 2005). The warped lesion map was then binarized with a 50% probability threshold, which was used to perform voxel-wise analyses.

For each model estimate, we ran a whole-brain voxel-based lesion-parameter mapping analysis using SVRLSM (DeMarco & Turkeltaub, 2018). Images were resampled to 4mm$^3$ isotropic voxels. We corrected for lesion volume by regressing it out of both behavior and lesion data and included only voxels with damage in at least 8 participants (10.1%; coverage shown in Fig. 3A). Resulting voxel-wise SVR-β values were thresholded at p<.005 based on 10,000 permutations. In the resulting thresholded statistical maps, we used the connected$\_$components method in nilearn (Abraham et al., 2014) to extract coordinates and volume for groups of suprathreshold voxels (Table 2). Only clusters with a volume of 3,200mm$^3$ (i.e., 50 contiguous 4mm$^3$ voxels) or greater are shown in the table.

**Table 1:** Proportion of variance (predicted R$^2$) in observed response type rates recovered by the fitted models. All values p<0.01, Bonferroni corrected for multiple comparisons

	Correct	Hierarchical	Linear	Mixed	Omission
MPT	0.995	0.975	0.968	0.815	0.997
Multinomial	0.999	0.998	0.995	0.985	0.988

**Fig. 2:**
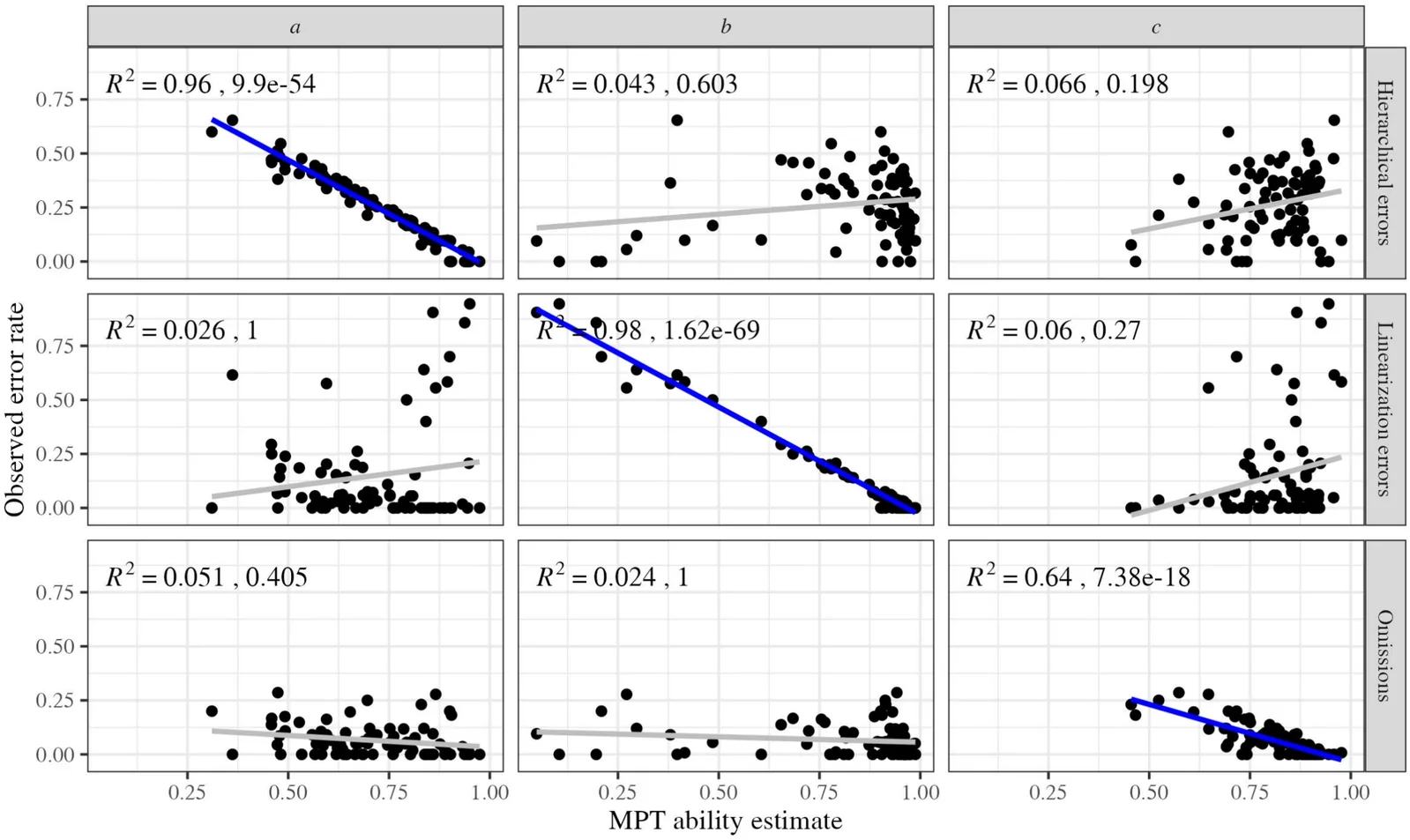
Correlations between MPT ability estimates (*x*-axis) and observed behavior (*y*-axis). *Blue lines* indicate significant negative correlations and *gray lines* show non-significant relationships. $R^2$ and *p* values for each correlation are shown. *P* values are Bonferroni-corrected for multiple comparisons at p<0.01

## Results

### Model parsimony with behavioral data

To evaluate point estimates, we examined the proportion of variance in observed response type rates that could be recovered from the fitted model. We examined response type rates instead of frequencies, because partial information about variation in response type frequencies across participants is provided to the model in the form of the total number of sentences observed. By contrast, estimated response type rates are entirely a product of estimated latent variables. The proportion of variance in each response type rate that could be recovered with the fitted models is shown in Table 1. Although the MPT model was less accurate when recovering Mixed error rates, the average proportion of variance in response rates that could be recovered with the fitted model across all response type rates was .936. These results indicate that the observed data was mostly consistent with the fitted MPT model’s posterior predictions.

The MPT model had a DIC of 1284 while the multinomial model had a DIC of 1154 (where lower DIC indicates the preferred model). These results indicate that the MPT model had an unexpected lack of information about response type rates, particularly mixed errors, given its degrees of freedom.

We found that the MPT ability estimates correlated well with observed behavior (Fig. 2). In particular, we found significant negative correlations between ability estimates and observed error rates for the corresponding error types: *a* was negatively correlated with hierarchical error rate, *b* with linearization error rate, and *c* with omission error rate. This result is intuitive to interpret: individuals with higher abilities produce fewer errors. We also found significant positive correlations between ability estimates *a* and *b* and linearization and hierarchical error rates, respectively.

**Fig. 3:**
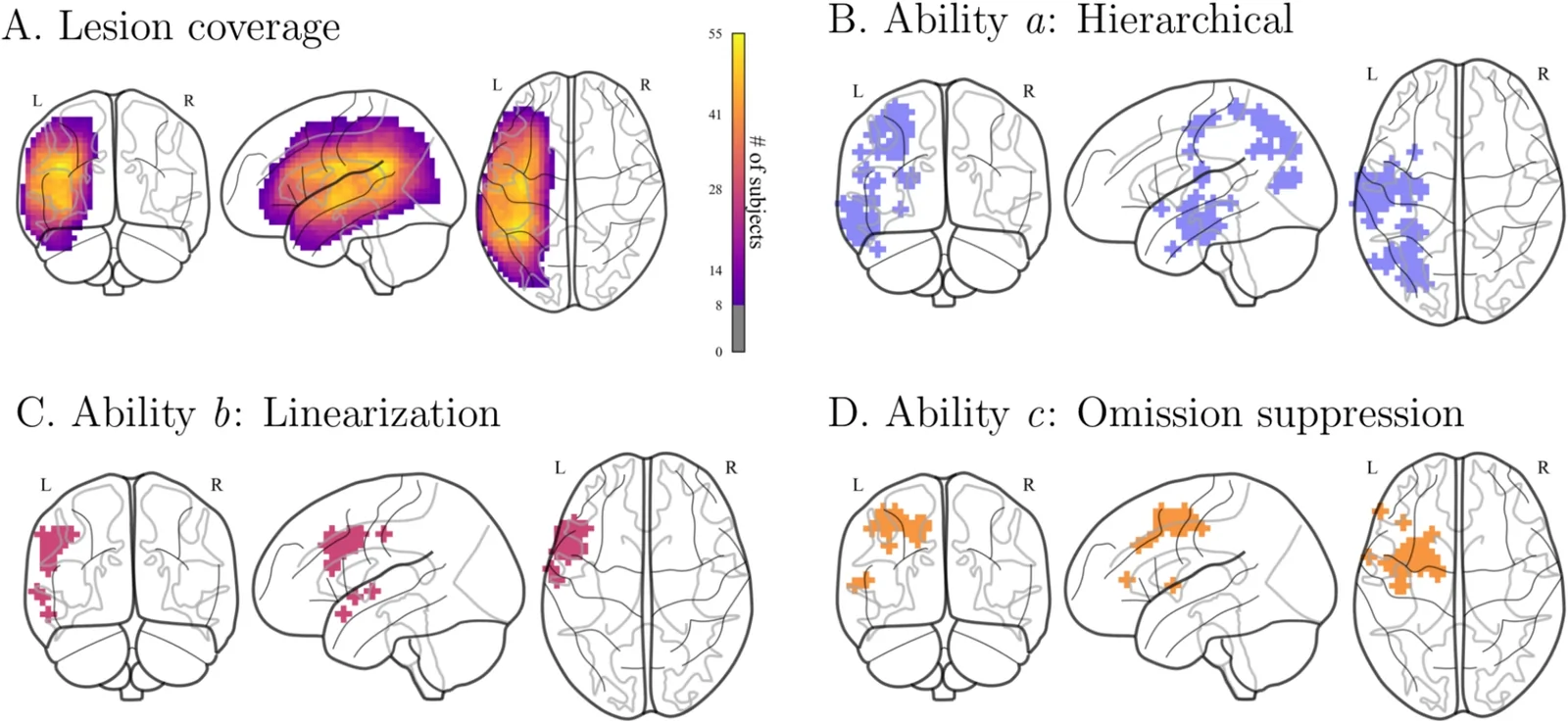
Neuroimaging results. **A** Lesion overlap showing voxels included in the analysis with damage in at least eight participants. **B** Thresholded lesion map associated with ability *a* (probability of successful hierarchy building). **C** Thresholded lesion map associated with ability *b* (probability of successful linearization). **D** Thresholded lesion map associated with ability *c* (Probability of a given error presenting as a single morpheme omission)

**Table 2:** Coordinates in MNI space and volume for connected components of lesion correlates of model estimates

**Model**	**Estimate**	**X**	**Y**	**Z**	**Size (mm** $^3$ **)**	Region
MPT	*a*	-58.25	-15.02	-16.11	10,176	Medial MTG
MPT	*a*	-40.87	-63.52	-47.55	4288	Angular gyrus
MPT	*b*	-52.53	16.94	34.69	6272	MFG/IFS
MPT	*c*	-32.04	-0.91	49.97	9024	SFS
Multinomial	Hierarchical	-29.01	-78.26	19.97	4416	Inf Par/MOG
Multinomial	Mixed	-64.99	-14.75	-9.16	4288	Medial MTG
Multinomial	Mixed	-27.81	-77.44	23.40	5504	Inf Par/MOG
Multinomial	Linearization	-50.84	12.91	29.3	18,944	IFG/vPrCG

*MTG* middle temporal gyrus, *MFG* middle frontal gyrus, *IFS* inferior frontal sulcus, *SFS* superior frontal sulcus, *Inf Par* inferior parietal, *MOG* middle occipital gyrus, *IFG* inferior frontal gyrus, *vPrCG* ventral precentral gyrus

For correlations between the behavior predicted by the two fitted models and the observed behavior, we found that both models fit the observed behavior very well, with all $R^2 \ge 0.82$, and all values apart from Mixed errors under the MPT model $R^2 \ge 0.97$. The multinomial model produced higher $R^2$ values than the MPT model overall.

### Lesion correlates of model estimates

Despite the MPT model’s lower DIC relative to the multinomial model, the theoretical structure of its parameters (each corresponding to a specific stage of the production process) generates clear, directional predictions about their neural correlates that the multinomial model’s parameters do not. We therefore examined the lesion correlates of the MPT ability estimates as a test of whether the parameters capture theoretically meaningful variation in processing, independent of their raw fit to the behavioral data (Roberts & Pashler, 2000).

We found that the ability estimate *a* (i.e., probability of successfully building the hierarchical structure of the intended utterance) corresponded to damaged voxels in the middle temporal gyrus (MTG), and in the posterior inferior parietal lobe, including the angular gyrus (Fig. 3B; Table 2). Ability estimate *b* (i.e., the probability of successfully linearizing a hierarchical structure; Fig. 3C) corresponded to damaged voxels in the posterior frontal lobe, especially the middle frontal gyrus (MFG) and inferior frontal sulcus (IFS). Ability estimate *c*, which corresponds to the probability that some error in either hierarchy or linearization will present as an omission (Fig. 3D) was associated with damage to voxels in the superior frontal sulcus (SFS) and underlying white matter extending under the dorsal precentral gyrus.

We further examined the lesion correlates of the error commission probabilities derived from the multinomial model (Table 2). Lesions associated with the model estimates for hierarchical and mixed errors both implicated regions in the inferior parietal lobe and middle occipital gyrus (MOG). Mixed errors also implicated the medial MTG. Linearization errors implicated a region encompassing most of the inferior frontal gyrus (IFG) and parts of the ventral precentral gyrus. No regions were associated with omission errors.

## Discussion

We have presented a novel Bayesian multinomial processing tree (MPT) model to characterize syntactic error commission in individuals with chronic post-stroke aphasia, representing the first computational instantiation of a two-stage model of sentence production (Bock & Levelt, 1994; Ferreira et al., 2018). The goal of this work was to apply the MPT modeling framework to syntactic error commission to provide a theoretically and mechanistically motivated computational model of sentence production errors, following prior work applying MPT models to picture naming errors (e.g., Walker et al., 2018; Walker et al., 2024). Crucially, this approach moves beyond holistic clinical impressions of a patient’s speech to derive quantitative, stage-specific ability estimates for each individual: estimates that characterize *where* in the production process a given patient’s deficit lies, rather than simply describing the surface-level distribution of their errors.

We also carried out lesion-parameter mapping to probe whe-ther the parameter estimates produced by the model might advance our understanding of functional neuroanatomy. This approach has previously been used to map the parameters derived from models of single word production to their lesion correlates (Dell et al., 2013). Whereas Dell et al. (2013) were mapping the lesion-parameter correlates of the dual-route interactive two-step model to explain observed single word production errors, our model aims to implement the HiLine model of syntactic production to explain observed ungrammatical sentence productions. Both models suppose that observed utterances (single words or multi-word utterances/sentences, respectively) rely on a sequence of multiple upstream processes to be produced correctly. Dell et al. (2013) found that the lexical-semantic and lexical-phonological parameters derived from their model had lesion correlates consistent with previous findings from the literature, and used this to inform their support of the dual-route model.

Similarly, the ability estimates generated by our MPT model had meaningful lesion correlates that closely replicate and extend prior lesion-symptom mapping studies of syntactic deficits (Casilio et al., 2024; Matchin et al., 2020; Yeaton et al., 2026). Hierarchical encoding ability (*a*) was associated with damage to the posterior superior temporal sulcus and angular gyrus, consistent with proposals that the posterior temporal lobe supports the construction of hierarchical syntactic structure (Hickok, 2025; Matchin & Hickok, 2020). Linearization ability (*b*) was associated with damage to the posterior frontal lobe, including the middle frontal gyrus and inferior frontal sulcus, consistent with proposals that frontal regions support the sequential implementation of syntactic structure (Bock & Levelt, 1994; Matchin & Hickok, 2020). That the MPT parameters, derived purely from behavioral error distributions, localize to theoretically predicted neural regions provides convergent validation of both the model’s theoretical structure and the underlying two-stage account of grammatical encoding.

We found that although our MPT model captured a substantial proportion of the variance observed in our data, a simpler multinomial model had a lower DIC, indicating better fit relative to model complexity. However, we argue that this comparison does not undermine the value of the MPT approach, for two reasons (Roberts & Pashler, 2000). First, model fit and parameter interpretability are distinct virtues. The multinomial model’s parameters are simply restatements of the observed error rates – knowing that a patient has a linearization error rate of 0.3 adds nothing beyond the raw data. By contrast, the MPT model’s parameters represent latent processing abilities that have a theoretical interpretation grounded in the two-stage architecture: knowing that a patient has low *b* (linearization ability) but intact *a* (hierarchical ability) characterizes their deficit in a way that is both mechanistically meaningful and potentially clinically actionable. Second, the meaningful and theoretically predicted lesion correlates of the MPT parameters – which are not guaranteed by the fitting procedure – provide independent validation that the parameters are capturing something real about the underlying processing stages, rather than being arbitrary decompositions of the error distribution.

Our result may nonetheless reflect genuine limitations of the current model formulation. Although mixed errors are coded as a uniform group, they appear to be more complex than a coincidental co-occurrence of hierarchical and linear errors. Furthermore, omission error rates may interact with the rates of other error types rather than being a fixed, independent probability. The relatively low frequency of mixed (n=146) and omission (n=215) errors compared to grammatical ($n = 1{,}792$), hierarchical (n=707), and linearization (n=279) utterances likely exacerbates these issues, as parameter estimates for rarer categories rely more heavily on the prior distribution and are therefore less constrained by the data. Future work could address this by collecting larger discourse samples, by refining the treatment of Mixed errors (e.g., by distinguishing cases where hierarchical and linearization deficits co-occur from cases where a single underlying deficit produces surface features of both) or by exploring richer model architectures that allow the omission parameter to vary as a function of error type rather than being fixed across all error-producing pathways.

Several limitations of the present study should be noted. The model was fit to discourse samples from a single elicitation task (Cinderella story retelling), and it remains to be seen whether the parameter estimates generalize across different discourse genres or more structured production tasks. Additionally, the moderate interrater reliability for hierarchical errors reported in Yeaton et al. (2026) (consistent with the broader difficulty of reliably identifying paragrammatic features Matchin et al., 2020) introduces some noise into the input data, particularly for the hierarchical error category. Future work with refined coding criteria or automated coding approaches could reduce this source of variability.

Overall, the MPT model presented here makes three contributions. First, it constitutes the first formal computational implementation of a two-stage model of sentence production, demonstrating that such a model can be instantiated as a generative process and fit to behavioral data. Second, it provides a principled method for deriving individual-level, stage-specific ability estimates from naturalistic discourse samples – a tool that could inform both clinical profiling of syntactic deficits and neurolinguistic investigations of the sentence production system. Third, the convergence of the model’s lesion correlates with those predicted by the HiLine framework provides the strongest computational evidence to date that hierarchical and linearization stages of grammatical encoding are neurally dissociable. Taken together, these findings bring the analysis of syntactic errors in line with more established model-based error analysis methods in aphasia research (Walker, 2021; Walker et al., 2018), and open the door to more fine-grained computational accounts of the syntactic production system in both healthy and disordered language users.

## Data Availability

The de-identified data are available as electronic supplementary material.

## Code Availability

The code for the model is available as electronic supplementary material. Code and de-identified data are in an OSF repository here: https://doi.org/10.17605/OSF.IO/5WTX6.

## Declarations

### Open Practices Statement

The data and code for the model are available as electronic supplementary material. The study was not preregistered.
